# Comparative institutional analysis for public health: governing voluntary collaborative agreements for public health in England and the Netherlands

**DOI:** 10.1093/eurpub/cky158

**Published:** 2018-11-01

**Authors:** Marleen P M Bekker, Nicholas Mays, Jan Kees Helderman, Mark Petticrew, Maria W J Jansen, Cecile Knai, Dirk Ruwaard

**Affiliations:** 1 Department of Health Services Research, Care and Public Health Research Institute, Faculty of Health, Medicine and Life Sciences (CAPHRI), Maastricht University, Maastricht, The Netherlands; 2 Chair group Health and Society, Center for Space, Place and Society, Wageningen University and Research, Wageningen, The Netherlands; 3 Policy Innovation Research Unit, Faculty of Public Health and Policy, London School of Hygiene and Tropical Medicine, London, UK; 4 Department of Public Administration, Institute for Management Research, Radboud University, Nijmegen, The Netherlands

## Abstract

Democratic institutions and state-society relations shape governance arrangements and expectations between public and private stakeholders about public health impact. We illustrate this with a comparison between the English Public Health Responsibility Deal (RD) and the Dutch ‘All About Health…’ (AaH) programme. As manifestations of a Whole-of-Society approach, in which governments, civil society and business take responsibility for the co-production of economic utility and good health, these programmes are two recent collaborative platforms based on voluntary agreements to improve public health. Using a ‘most similar cases’ design, we conducted a comparative secondary analysis of data from the evaluations of the two programmes. The underlying rationale of both programmes was that voluntary agreements would be better suited than regulation to encourage business and civil society to take more responsibility for improving health. Differences between the two included: expectations of an enforcing versus facilitative role for government; hierarchical versus horizontal coordination; big business versus civil society participants; top-down versus bottom-up formulation of voluntary pledges and progress monitoring for accountability versus for learning and adaptation. Despite the attempt in both programmes to base voluntary commitments on trust, the English ‘shadow of hierarchy’ and adversarial state-society relationships conditioned non-governmental parties to see the pledges as controlling, quasi-contractual agreements that were only partially lived up to. The Dutch consensual political tradition enabled a civil society-based understanding and gradual acceptance of the pledges as the internalization by partner organizations of public health values within their operations. We conclude that there are institutional limitations to the implementation of generic trust-building and learning-based models of change ‘Whole-of-Society’ approaches.

## Introduction

In public health policy, alternative types of social coordination between the classical hierarchical state, the market and civil society have emerged since the 1970s.^1–3^ These have been characterized as a move ‘from government to governance’.^4–6^ In contrast to the old state-centric theory that focussed merely on unilateral governmental ‘steering’ through regulation, command-and-control and public service provision, which is to an important extent still the default mode in public health policies, modern governance theories take a wider view of the whole repertoire of modes of social coordination for collective action by state *and* society,^5^^,^^7^^,^^8^ and how these have been institutionalized in governance arrangements.^8^^,^^9^ How public health policies converge or diverge across countries over time can be analyzed and explained using comparative institutional analysis, thereby clarifying under which institutional conditions an alternative governance mode for public health is likely to achieve its goals.^10^^,^^11^

Due to different institutional starting conditions, welfare states have developed different state-society relations over time, resulting in different governance arrangements. For instance, the majoritarian democracy of the United Kingdom produces adversarial and short-term state-society relationships because a change of government can produce sharp ideological shifts in policy. To retain continuity of public administration in such situations, the United Kingdom has a hierarchical and relatively centralized government.^12^^,^^13^ In contrast, in some continental European countries, governance emerged out of generally devolving public tasks to semi-public or publicly licensed private not-for-profit organisations, thereby setting the foundations for distinct welfare state governance arrangements.^14^^,^^15^ In the Netherlands, this is known as the ‘poldermodel’—a system of consensus-based democracy that allows for longer term relationships between state and society, and the gradual uptake and modification of new ideas over time.^16^

In this article, we show how these different institutional contexts and governance arrangements have shaped the developmental trajectories of two apparently similar public health governance programmes: the English Public Health Responsibility Deal (RD) and the Dutch ‘All About Health…’ (AaH) programme. The perceived success or failure of the programmes in terms of their impacts such as on behaviour and health falls beyond the scope of this article.

### Towards a new social contract between state and society

In 2010, the World Health Organization (WHO) issued the Adelaide Statement proposing a new ‘social contract’ that has become known as the ‘Whole-of-Society’ (WoS) approach, aimed at inducing both public and private actors (governments, civil society and business) to take responsibility for the co-production of economic utility and good health.^1^^,^^3^ WoS approaches typically reflect collaborative governance arrangements and platforms across state and society, bringing different capacities together, enabling tailored solutions and offering space for early conflict resolution.^17^^,^^18^ One manifestation of this is the establishment of public–private partnerships (PPPs) and voluntary agreements designed to improve public health, either in lieu of state regulation or as a supplement.

The English Public Health RD and the Dutch AaH programmes are two recent examples of this phenomenon with very similar goals. In both cases, the notion of the ‘social contract’ is manifest through use of so called ‘pledges’. These are presented as voluntary agreements whereby non-state actors express their commitment to public health improvement goals. While the Dutch programme was extended in 2016 for five more years to 2021,^19^ the English programme was quietly wound down by government from 2015.

Why was this so? Since neither programme met the presumed requirements for effective voluntary agreements identified from the literature,^20^ this cannot explain their different trajectories. Instead, we look to the way in which each programme and its supporting governance arrangements relate to its institutional context.^10^

## Methods

A ‘most similar cases’ design^11^^,^^21^ allows us to understand the way institutions shape novel policy and coordination initiatives such as the RD and AaH. We conducted a qualitative comparative secondary analysis of data from the evaluations of the two programmes to explain the similarities and differences between two programmes with seemingly very similar goals. Each evaluation included a literature review, analysis of pledges and public documents, and qualitative interviews (44 interviews with 50 individuals plus six qualitative organizational case studies in the RD study; 85 interviews in the AaH study) with purposive samples of direct participants such as government officials, programme implementers and pledge signatories, and non-participant stakeholders in the public and private sectors. In both studies, interviews covered: aims, objectives and activities; pledges and network development; motivations for participation; experienced barriers and facilitators; perceived strengths and weaknesses; necessary improvements including requirements for sustainable action; costs and achievements; and future expectations.^22^^,^^23^

## Results

### Characterizing the two programmes

#### English public health responsibility deal

The RD was launched by the Conservative-Liberal Democrat Coalition Government in 2011 as a PPP between government, business, the public sector and non-governmental organizations (NGOs) to improve public health in the areas of food, alcohol, health at work and physical activity.^24^ ‘Five core commitments’ defined the scope, purpose and ambitions of the RD, which all partners had to make (see table 1).

**Table 1:** ‘Five core commitments’ defining the scope, purpose and ambitions of the RD

RD	Core commitments
1.	We (business, NGOs and public bodies joining the RD) recognize that we have a vital role to play in improving people’s health.
2.	We will encourage and enable people to adopt a healthier diet.
3.	We will foster a culture of responsible drinking, which will help people to drink within guidelines.
4.	We will encourage and assist people to become more physically active.
5.	We will actively support our workforce to lead healthier lives (Department of Health, 2011).

Andrew Lansley, then Secretary of State for Health, asserted that,

By working in partnership, public health, commercial, and voluntary organisations can agree practical actions to secure more progress, more quickly, with less cost than legislation. ……., we shouldn’t be scared to use the reach of businesses to achieve mutually beneficial aims. Put simply, commercial organisations can reach individuals in ways that other organisations, Government included, cannot.^24^ (p. 2).

The RD was overseen by a Plenary Group (chaired by the Secretary of State for Health), and consisted of four networks chaired by either topic experts or participants in the sector: Food, Alcohol, Health at Work and Physical Activity. Each network had Steering and Working groups consisting of representatives of participating sectors, and developed collective pledges (see table 2). Partners were required to sign to at least one collective pledge from any of the networks, could develop individual pledges and had to produce delivery plans as well as annual progress reports. The Department of Health (DH) placed these on a dedicated part of its website^.24^ By March 2016, 776 organizations had pledged to participate in the RD.

**Table 2:** Examples of RD collective pledges across the four networks launched in March 2011

Name		Why a pledge	Who is involved	What activities	More info
	Food Network Collective pledge:F1: Out of home calorie labelling	‘Out of home calorie labelling aims to inform and empower people to make healthier choices more often when eating out, as well as encouraging food businesses to make healthier options more available’.	Catering businesses, who sell food in out of home settings; including supermarkets, fast food companies, cafes 42 signatories with a pledge delivery plan 5 signatories without plan	‘We will provide calorie information for food and non-alcoholic drink for our customers in out of home settings from 1 September 2011 in accordance with the principles for calorie labelling agreed by the Responsibility Deal’.	http://webarchive.nationalarchives.gov.uk/20180201180712/https://responsibilitydeal.dh.gov.uk/f1-factsheet/
	Alcohol network collective pledge: A1: Alcohol labelling	‘The …collective pledges support the core commitment to foster a culture of responsible drinking, which will help people to drink within guidelines’.	Alcohol producers: ‘This pledge commits alcohol producers to label their products with unit and health information’. 81 signatories with a pledge delivery plan 12 signatories without a plan	‘We will ensure that over 80% of products on shelf (by December 2013) will have labels with clear unit content, NHS guidelines and a warning about drinking when pregnant’.	http://webarchive.nationalarchives.gov.uk/20130104160317/http://responsibilitydeal.dh.gov.uk/2012/11/15/public-health-responsibility-deal-collective-pledges/
3	Physical Activity Network collective pledge P1: Physical activity: Community	To encourage and assist people to become more physically active; ‘A healthy, active population is good for business and the economy as a whole’.	Business, voluntary, community and other organizations 99 ‘organizations committed to this pledge’	‘We will use our local presence to get more children and adults more active, more often including engaging communities in planning and delivery’.	http://webarchive.nationalarchives.gov.uk/20180201181151/https://responsibilitydeal.dh.gov.uk/pledges/pledge/? pl=17
4	Health at Work Network collective pledge H3: Health and wellbeing reports –	‘This pledge will raise the profile of employee health and wellbeing and ensure this issue is integral to all organizations’.	Employers large- and small-scale businesses, public and social organizations, governmental organizations 261 ‘organizations committed to this pledge’	‘We will include a section on the health and wellbeing of employees within annual reports and/or websites. This will include staff sickness absence rates’.	http://webarchive.nationalarchives.gov.uk/20180201181435/https://responsibilitydeal.dh.gov.uk/pledges/pledge/? pl=13

See: http://webarchive.nationalarchives.gov.uk/20180201175731/https://responsibilitydeal.dh.gov.uk/wp-content/uploads/2012/03/The-Public-Health-Responsibility-Deal-March-20111.pdf.

#### The Dutch ‘All About Health…’ programme

At the request of Parliament, the Ministry of Health, Welfare and Sport (HWS) developed a National Prevention Programme (NPP) in 2012 to connect the ‘predominantly small-scale, fragmented and ad hoc health initiatives in society’, and increase collaboration, scaling up and focus^25^ (p. 4). It comprises existing legislation as well as government-led health programmes and a newly added ‘health movement’, called AaH, in which societal partners pledge to take initiatives to achieve public health goals.

The long-term goals of the NPP are to reduce chronic conditions (such as diabetes and depression) as well as smoking, alcohol abuse, physical inactivity and obesity, and to counter growing health disparities. Within the NPP, AaH, using a bottom-up approach aims to help develop a ‘social movement for a healthier and more vital Netherlands’^25^ (p. 8), inviting societal partners with inspiring health initiatives to engage with it. Partner commitment to the AaH is via a ‘pledge’: ‘a public statement by which an organisation expresses commitment and an active contribution to the realization of governmental health goals by conducting specific activities.’ (www.allesisgezondheid.nl) Pledges do not impose any mandatory requirements and partners can formulate their own pledges. ‘Each partner is responsible for the activities and results in their own domain, can be peer reviewed by other partners, and will be publicly held accountable’^25^ (p. 16). By January 2018, 374 pledges had been signed by 684 organizations with another 2200 organizations involved in the domains of the neighbourhood, school, work, healthcare and health protection. Of the pledges, 63% are collaborative, involving multiple partners across different domains.^26^^,^^27^ The Ministry of HWS funds a small Programme Office, publishing the signing of pledges on social media, acquiring new partners and pledges, and organizing regular events for knowledge exchange. Progress is reported to Parliament at least once a year, informed by monitoring and evaluation reports from the Programme Office and independent academic research commissioned by the Netherlands Organization for Health Research and Development (ZonMw).

See table 3 for examples of pledges.

**Table 3:** Examples of collaborative pledges in the Dutch AaH programme, (serving as case studies in the evaluation study 2015–2016)

Pledge name/initiator		Why a pledge	When initiated	Who is involved	What activities	More info (in Dutch)
1	Deltion school for vocational training (15.000 students and 1500 staff)	Vitality in org. mission statement Cross-org. work group Integrated Health Management: ‘sustainable labour participation’	Pledge signed on 10 April 2015 IHM work group initiated 10 years ago	Initiators: Sports Expertise Centre, Human Resources Development director Other members IHM: educational managers, labour expert	First year students Vital Citizenship program (dit test, test lifestyle, vitality education, sports) continued by electives on vitality in various vocational contexts (e.g. transport and logistics)	https://www.deltion.nl/vitaliteit
2	Network ‘Positive Health’ Northern Maasvallei	Promoting ‘Positive Health’ concept among all care professionals in region	11 April 2014	Health and social care providers, municipalities and Positive Health Institute	Positive Health Screening and Conversation tool, restructuring organization and funding primary and social care	http://www.netwerkpositievegezondheid.nl/
	Care Innovation Centre W-B	Access to and advice on care innovations and user feedback, enhance prevention and self-care at home	13 November 2014 as well as regional network coordinating > 40 local pledges	Vocational school, product developing industry, municipalities, clubs for the elderly, home care providers	Physical, digital and mobile ‘House of Tomorrow’ display, advice and try out of new products; process and system innovations pilot	https://www.cic-westbrabant.nl/
	Stay Clear campaign (Heineken and NOC-NSF Sports Association)	Unbranded Promotion of ‘responsible drinking’ sports club canteens = no alcohol <18, and responsible drinking >18	22 May 2015 after being invited by the Programme Office	2016 500th sports club participating in the campaign; annual contest ‘Most clear sports club’	Creating awareness and health promotion; mystery visits by 18 year olds checking compliance, offering assistance; monitoring of change in sports canteens after second visit	https://www.blijfhelder.nl/
	AaH programme (Ministry of Health, Welfare and Sport)	Population health, participation of citizens with disabilities, Healthy employees	14 June 2014	Programme lead within Ministry = Executive Council (as of 2017 = DG Public Health)	Prevention in SIS^a^ healthcare policies, healthy human resources, research and monitoring, Health in all Ministry policies (prevention in healthcare)	https://www.rijksoverheid.nl/onderwerpen/gezondheid-en-preventie/nationaal-programma-preventie
	Alliance for Health and Literacy	Growth pledge improving literacy as a resource for health, 1, 3 mlln low literate citizens (8% Dutch population) with 1, 5 higher mortality risk.	4 February 2015 + governmental programme ‘Language for Life’	Lead: Foundation for Reading and Writing, 60 participating organizations in healthcare, social care, education, and medium and large-scale employers	Educate 15 000 volunteers as Language-for-life coaches Conduct 60 000 basic language assessments Refer 45 000 participants to trained Language-for-life coaches	https://www.lezenenschrijven.nl/wat-wij-doen/programmas/alliantie-gezondheid-en-geletterdheid/

a Social Insurance healthcare System, in which prevention takes shape of individually insurable products and services only.

### Similarities and differences between the RD and AaH programmes

A prominent similarity between the RD and AaH consists of the underlying rationale that voluntary agreements are better suited than regulation to engage business and civil society to take responsibility for health. Although both programmes were government initiated, they considered the non-state ‘ownership’ of deals and pledges as crucial, although neither has direct citizen representation in its governance. Both had low entrance and exit thresholds, without any sanctions for non-participation or non-compliance. Finally, both governments have reported progress publicly including to Parliament while making it clear that implementation of pledges is the responsibility of partner organisations.^19^^,^^28^^,^^29^

The analysis of stakeholder interviews in both evaluations identified similar motivations, experiences and reported achievements. In both programmes, non-health partners participated out of intrinsic as well as extrinsic motivations, such as: public visibility and recognition; acquiring status and legitimacy; and access to networks and knowledge. Partners in both programmes expected that their pledges would accelerate existing activities and health awareness, but they also voiced concern over pledge delivery and the benefits that were to be gained from programme participation.^30^^,^^31^

A third similarity is that both programmes encountered pressures, from political representatives, civil society or public health experts, to be more public-facing, to formulate more specific and measurable objectives, and to hold the pledge-partners accountable for their contributions to these objectives and overall public health goals.^29^^,^^30^^,^^32^^,^^33^ These pressures did not lead to significant changes in programme management.

One of the main differences between the programmes is that the RD promised ‘to secure more progress, more quickly, with less cost than legislation’^24^ (p. 2) by involving business, while AaH promised to connect existing yet fragmented initiatives in society and encourage new ones into a health movement, often referred to as ‘turning drops into a wave’^25^ (p. 8) that would be complementary to government action. This also generated distinctive expectations about the role of government in each programme, and about programme coordination. In the RD, the initial set of collective pledges was formulated in a relatively top-down way by Networks led by government-appointed representatives of industry before civil society organizations were invited to join. All pledges had to be approved not only by the Network chairs but also by the DH. This implied a certain degree of supervision and follow up, which was only partially adhered to.^30^ In AaH, the partners formulate their own pledges and mobilise their own local partners and networks. Government adhered to a facilitative role mainly.

This also highlights how the types of participants differed in the two programmes. In the RD, the private sector (often multinational businesses) dominated the Food and Alcohol Networks, with public sector organizations and NGOs only slightly more prominent in the Physical Exercise and Health at Work Networks. Ministers were actively involved in encouraging firms to join the RD and make pledges.^23^ Public health advocacy organizations felt compelled to refuse invitations to participate in the RD due to what they saw as conflicts of interest on the part of industry participants in the design of pledges.^14^^,^^18^ In contrast, during the initial interactive development phase of the AaH programme in 2012 and 2013, involvement of representatives from a wide range of societal interests created a broad level of tolerance, if not support, among stakeholders.^34^ A wide range of local, regional and national NGOs, many small and a few large businesses, and public sector organisations joined, some of these by invitation, others by their own initiative.

The most remarkable difference, however, is how the partners in both programmes interpret the processes of networking, formulating and implementing pledges, and monitoring progress. In the RD, participant interviewees reported ‘playing it safe’ and a substantial number of interviewees reported not doing anything particularly new or different to their usual practice as a result of the RD. The monitoring of activities against the pledges was almost entirely focused on demonstrating the scale and reach of the RD (e.g. the number of new pledges and organisations involved). Moreover, as time progressed, partners expected the Government to provide more leadership in order to ensure a ‘level playing field’ between partners, and between partners and non-partners, and to give public recognition for pledge-based achievements.^30^ Similar to the RD, AaH interviewees also articulated a need for more interaction with, and help and reward from, the Government including non-health Ministries.^31^ In response, the Ministry of HWS promised to ‘discuss and take measures to remove obstacles in laws and regulations’.^19^ Many AaH partners similar to the RD mentioned organizing pre-existing activities, yet, they also described how the pledges gave new impetus to implementing these pre-existing plans, and do more. AaH partners performed several governance tasks: some provided evidence, others contributed to policy development, exercised advocacy, helped consensus building, acted as watch dogs, provided services to members and to the public, acted as self-regulators and/or were key in industrial relations in the health sector.^35^ They described the pledge network processes as organic, pragmatic and adaptive to local feedback. Instead of jointly developing detailed project plans with specific, measurable, achievable, relevant and time-bound (SMART) objectives between local partner organisations, the pledge initiators conducted ad hoc activities in direct contact with target groups in order to keep the partners committed and not to lose partners through bureaucratic procedures. These activities are accompanied by a high degree of ‘reflection-in-action’ generating participant feedback and adapting activities accordingly, described by one initiator as ‘reflexive monitoring’ (interview 25 April 2016). The absence of specific pledge requirements created time and space for exploratory networks of potential partners seeking mutual benefits before deciding to engage in more entrepreneurial networks. The interview data in AaH seem indicative of a general enthusiasm for initiatives that the Programme Office frames as ‘disruptive innovation’, and which participants regard as ‘system-breakthroughs’ that remove barriers to change and create room for (social) innovation. In some pledge networks, this has boosted a felt urgency to reorganize organizational procedures, rules of engagement, the division of responsibilities and funding arrangements. These processes contributed to increased number of partners, domain-crossing pledge networks, awareness of the relevance of health in different settings and sectors, and identification of obstacles to innovation and new ways of working.^31^

## Discussion

The comparative institutional analysis has generated two partial, yet inter-related, explanations for the different compositions and trajectories of the programmes: the nature of the micro-level pledges and their formation; and the macro-level institutional context, more specifically, the types of democratic institutions and governance regimes in which the two programmes are embedded at the meso-level of policy systems.

### The nature of the pledges and their formation

Although both programmes rely on voluntary, trust-based pledges, there is an important difference in how the RD and AaH pledges were developed by government and perceived by the non-state partners. Signing a pledge reflects the more or less credible commitment of partners to health-related norms and values (awareness) and perhaps the social approval and reputation that they gain by doing so.^9^ We theorize that these micro-level pledges can be located on a continuum between, on the one hand, ‘voluntarily’ entered (quasi-) contracts establishing rights and obligations between actors with different resources and interests, and, on the other, entirely ‘trust-based’ commitments between actors. What matters for the exact location of the pledge on this continuum is the degree to which the public and private actors engaged in a pledge have *internalized* the promotion of good health (or the prevention of negative health externalities) into their own activities and strategic cost/benefit calculations in a credible way.

The RD pledges were perceived as ‘quasi-contractual’ relationships between government and other actors to achieve public health goals, notwithstanding the fact that government meant them to be ultimately trust-based since there was limited government oversight and no intention to resort to legislation. In AaH, pledges have continued to be treated as trust-based commitments by civil society actors towards (public) health goals. AaH seems to be flourishing in a context in which public and private partners have learned to acknowledge the fact that fast results cannot be expected and that the purpose of the programme is more about initiating a societal movement dedicated to the inclusion of health values and goals into the strategic choices that pledge partners make, rather than necessarily producing rapid, measurable results.

### The impact of the institutional context

The placing of voluntary pledges (at the micro-level of social exchange relations) on a continuum between quasi-contractual and entirely trust-based commitments fits the two different meso-level governance regimes and the macro-level democratic institutions. While the Netherlands developed a consensual democracy with employer and employee bodies co-formulating social policies with government, and a long history of private provision of public tasks and self-regulation, the United Kingdom depends on majoritarian democracy, with a structural lack of trust in government and adversarial public-private relations motivated by advocacy of, and resistance to, further privatization. Pledges in the hierarchical, relatively centralized, governance regime of the United Kingdom are likely to be regarded by non-governmental actors as more quasi-contractual in character (and critiqued accordingly for a lack of government supervision and control) while pledges in the more consensual governance regime of the Netherlands are likely to be perceived as more trust based. The RD and its pledges evolved primarily in the space between the private market (business) and central government, while in the Netherlands, the pledges were developed and agreed in the more or less organized civil society sphere.

Thus one explanation for the gradual, unobtrusive winding down of the RD from early 2015 and the continuation of the AaH until at least 2021 may lie in the way that the two programmes were developed and the role of central government in each process. In the RD, the prime objective of the government was to negotiate ‘deals’ with business and other actors that might improve public health. Although it was clearly hoped that the RD would become rooted in wider society, civil society organizations either refused to engage or withdrew on the grounds that it was based on a fundamental conflict of interest between business and public health imperatives. By 2015, the independent evaluation had cast considerable doubt on claims that the RD was achieving its health objectives and would be likely to contribute to significant improvement in public health. For example, the pledges tended to reflect activities that partners were already pursuing, or interventions unlikely to be effective.^30^^,^^36–41^

The AaH, on the other hand, was strongly associated with its initiating social actors and was more public facing. Also, the purpose of accountability was developmental and forward-looking rather than focusing summatively on goal achievement. The AaH Programme Office encouraged partners to be more ambitious, and publicly share experience with other partners. This approach is rooted in the more consensual political tradition of the Netherlands which goes some way to explain the more horizontal, ‘trust-based’ nature of AaH. This has enabled AaH to evolve toward a multi-lateral societal programme supported by a range of societal groups with strong participation of civil society.

## Conclusion

Comparative institutional analysis provides plausible explanations for the convergence or divergence between countries’ policies and trajectories, related to similarities or differences in their democratic institutions and governance regimes. The main lesson from the case for developing collaborative arrangements for public health is that the generic trust-building and learning-based models of change in WoS approaches require trust-generating institutional conditions. In their absence, programmes based on WoS principles could do more to create these conditions by implementing decentralised and small-scale initiatives and agreements embedded in local communities and face-to-face based networks.

### Funding

This report is based on independent research commissioned and funded by the NIHR Policy Research Programme via its core support for the Policy Innovation Research Unit (Project No: 102/0001). The views expressed in the publication are those of the authors and are not necessarily those of the NHS, the NIHR, the Department of Health and Social Care, its arm’s length bodies or other Government Departments.

The independent evaluation study of the AaH programme (2015–2017) was funded by the Netherlands Organization for Health Research and Development (ZonMw), project no. 531005010. A follow up study has been granted for 2018–2022, project no. 531 005012.

This open access Supplement received funding under an operating grant from the European Union’s Health Programme (2014–2020).

*Conflicts of interest*: None declared.

**Key points**

- The WoS approach of the WHO proposes a new ‘social contract’ aimed at inducing both public and private actors (governments, civil society and business) to take responsibility for the co-production of economic utility and good health.
- One manifestation of this approach are voluntary public–private agreements such as the Public Health RD in England and the AaH programme in the Netherlands.
- A systematic comparative analysis of similarities and differences in contextual pressures, and in actor networks and institutional settings between the programmes helps explain their different trajectories and draw lessons for an institutionally compatible programme design.
- The English ‘shadow of hierarchy’ and adversarial state–society relationships conditioned non-governmental parties to see the pledges as controlling, quasi-contractual agreements that were only partially lived up to, while the Dutch consensual political tradition enabled a civil society-based understanding and gradual acceptance of the pledges.
- The generic trust-building and learning-based models of change in WoS approaches require trust-generating institutional conditions. In their absence, programmes based on WoS principles could do more to create these conditions by implementing decentralized and small-scale initiatives and agreements embedded in local communities and face-to-face based networks.
